# Wildlife in climate refugia: Mammalian diversity, occupancy, and tiger distribution in the Western Himalayas, Nepal

**DOI:** 10.1002/ece3.9600

**Published:** 2022-12-08

**Authors:** Kanchan Thapa, Samundra Ambuhang Subba, Gokarna Jung Thapa, Karun Dewan, Bishnu Prasad Acharya, Dabal Bohara, Suman Subedi, Madhuri Thapa Karki, Bharat Gotame, Gautam Paudel, Shiv Raj Bhatta, Shant Raj Jnawali, Sabita Malla

**Affiliations:** ^1^ WWF Nepal Kathmandu Nepal; ^2^ Division Forest Office Dadeldhura Nepal; ^3^ Ministry of Forests and Environment Kathmandu Nepal; ^4^ Department of Forests and Soil Conservation Kathmandu Nepal

**Keywords:** activity pattern, microrefugia, multi species occupancy model, occupancy, tiger

## Abstract

Anthropogenic land‐use change continues to be predicated as a major driver of terrestrial biodiversity loss for the rest of this century. It has been determined that the effect of climate change on wildlife population will accelerate the rate and process of decline of global vertebrate populations. We investigated wildlife composition, occupancy, and activity pattern along the larger climate resilient forests that serve as microrefugia for a wide range of species under the escalating climate change. We used camera trap survey covering 250 km^2^ of climate microrefugia in Dadeldhura hills in far western region of Nepal. We used 62 trapping locations accumulating 1800 trap nights taking 98,916 photographs in 62 days‐survey period during the summer season of 2020. We photographed 23 mammalian species with estimated species richness of 30 species (95% CI: 25–34) based on multi‐species occupancy model. We estimated overall species occupancy ψ(SE(ψ)) to be 0.87 (0.09) in climatic microrefugia. While human activity predominated throughout the day, the majority of animals was found to exhibit nocturnal temporal patterns. Tiger and hyaena, two of the top predators, were newly discovered in the western Himalayan range of Nepal, with their discovery at the 34 highest elevations of 2511 meters and 2000m, respectively. In Nepal, high‐altitude tiger range is characterized by tiger distribution above a 2000 m cutoff representing habitats in the physiographic zone of high mountains and above. Our findings establish a baseline and show that the climatic microrefugia that have been identified have high levels of species richness and occupancy, which characterize the Dadeldhura hill forest ranges as biologically varied and ecologically significant habitat. These areas identified as climatic microrefugia habitats should be the focus of conservation efforts, particularly efforts to reduce human disturbance and adapt to climate change.

## Graphical abstract

The paper describes wildlife community species richness, occupancy, and activity pattern in climatic microrefugia in the western Himalayan range of Nepal.

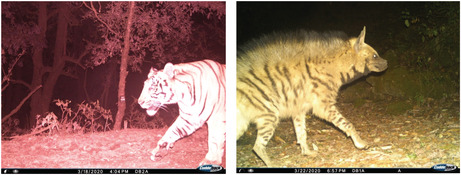

## 1 INTRODUCTION

Anthropogenic‐driven land‐use change has caused habitat loss, which has led to the highest rate of population reduction and is expected to continue as a key factor in the loss of terrestrial biodiversity for the rest of this century (Sala et al., 2000). Global vertebrate species population has decreased by an estimated 68% in the last 50 years (WWF, 2022). Climate change impact on species is not yet adequately documented by International Union for Conservation of Nature's (IUCN) Red List of Threatened Species (Akcakaya et al., 2006). However, climate change issue has been directly implicated in the deteriorating status of several vertebrates and may intensify the rate and process of ongoing drivers of decline to hasten extinction (Hoffmann et al., 2010; Laurance & Useche, 2009). It is estimated that 47% of threatened land mammals on the IUCN's Red List of Threatened Species have been negatively affected by climate change (Pacifici et al., 2017). Failing to address threats associated with climate change may further aggravate overall pressure to the biodiversity (Harfoot et al., 2021).

Understanding how drivers of change are interacting and impacting populations, and how this varies spatially, is critical if we are to identify populations at risk, predict species' responses to future environmental changes and produce suitable conservation strategies (Williams et al., 2022). Global Climate Risk Index 2021 ranks Nepal as the tenth most vulnerable country globally between 2010 and 2019 (Eckstein & Schäfer, 2021) based on extreme weather events and indicating a high level of exposure and vulnerability. Thapa et al. (2016) identified larger climate resilient forests as macrorefugia (including microrefugia within it) that needs to be conserved to limit the impacts of rising global temperatures in the 21st century (Ashcroft, 2010; Rull, 2009) for providing refuge to a wide range of species (small to large). Refugia are habitats that components of biodiversity retreat to, persist in and can potentially expand under changing environmental conditions (Keppel et al., 2012) triggered by climate change. Often, these habitats are also at risk of local species extinction from ongoing threats (Green et al., 2019) and future climate vulnerabilities (Bellard et al., 2012; Leclerc et al., 2020). Thus, understanding the pattern and drivers of species distribution and abundance is important for species conservation planning perspective under changing environmental conditions.

Mammalian diversity across the protected areas system is among the highest in South Asia which represents approximately 9.4% of the world's mammalian diversity (Srinivasulu, 2019). Existing large forest connectivity joining the impeding protected areas provides space for species dispersal, colonization, and recovery along the restored habitat (Green et al., 2019). Less empirical information is available on wildlife composition, population, and its distribution from area outside the protected areas which are a mix of forests, agriculture lands, and settlements in Nepal. The Churia, also called the Siwalik in India, and Mahabharat ranges are the youngest and highest mountain ranges, beside snowcapped Himalayas in Nepal. Thapa and Kelly (2016, 2017) carried out first detail systematic survey of wildlife in the Nepal's forgotten tiger land‐Churia‐ within the protected areas. Ecological relevance of Churia habitat for tiger conservation (Thapa & Kelly, 2017) contributed to nationwide survey of Churia physiographic zone for faunal assessment (Lamichhane et al., 2021). It is often argued that climate change and human disturbances are anticipated to alter both wildlife distributions and their movement patterns, increasing the risk of defaunation and habitat destruction for many endangered species and ecosystem (Gaynor et al., 2018; Li et al., 2019). Wild animals co‐occur with humans, animals may minimize risk by separating themselves in time rather than in space (Kronfeld‐Schor & Dayan, 2003). Temporal partitioning is a common, even basic phenomenon shaping spatiotemporal patterns of predation and competition (Gaynor et al., 2018). No research so far has been conducted on composition of wildlife communities, temporal pattern of wildlife, and potential species distribution—particularly mammals—in the targeted habitat identified as macro climate—refugia (Thapa et al., 2016) which are distributed along these physiographic zones‐lowland areas, Churia, middle mountain, and high mountain. Camera trapping, as employed here, provides an added opportunity to gain insight into species diversity (Rahman et al., 2021) and activity of Asian forest fauna that are difficult to see or are rare (Lynam et al., 2013).

In South Asia, tigers (*Panthera tigris tigris*) and leopards (*Panthera pardus fusca*) are found to occupy a wide range of habitats including alluvial floodplain grasslands, seasonally dry subtropical deciduous forests in the lowlands (Odden et al., 2010), the Bhabar (Thapa et al., 2014), the Churia (Thapa & Kelly, 2017), stretching beyond the subtropical into temperate areas up to alpine regions in the Himalayas for leopards (Wang & Macdonald, 2009) and mangroves deltas for tigers in the Sundarbans (Loucks et al., 2010). Majority of identified climatic‐refugia sites are either contiguous within species range (*insitu refugia*) and/or at distant to existing tiger habitat (*exsitu refugia*) in Nepal. Hence, with climate change impacts and tiger population on the rise in Nepal, tigers are known to be dispersing into newer habitats (Wikramanayake et al., 2004). Tigers require a functional ecosystem and climate resilient landscapes with undisturbed large tracts of habitat with enough prey for maintaining viable population for their long‐term survival (Wikramanayake et al., 2004). Understanding their presence and distribution along the habitat in high elevational range beyond its known range is crucial. Our objectives were to (1) quantify mammalian occupancy and richness in the climatic microrefugia site, (2) determine the temporal activity pattern of recorded mammalian species, and (3) assess the tiger distribution along the habitat of the high elevation gradient in Nepal. We used Bayesian analytical platform‐multi‐species occupancy model (MSOM)—an extension to the Single Species Occupancy Model, to assess presence, community composition, and species richness of mammals in climatic microrefugia site in Western Nepal (Iknayan et al., 2014; Kéry & Royle, 2020). This is the first attempt to quantify estimates of mammalian richness and occupancy, as well as the intrinsic temporal pattern of wildlife in the western landscape, and it provides important insight into what actions are required to conserve wildlife in an area outside the protected areas that are likely to be more resilient to climate change in the future.

## 2 METHODOLOGY

### 2.1 Study area

**FIGURE 1:**
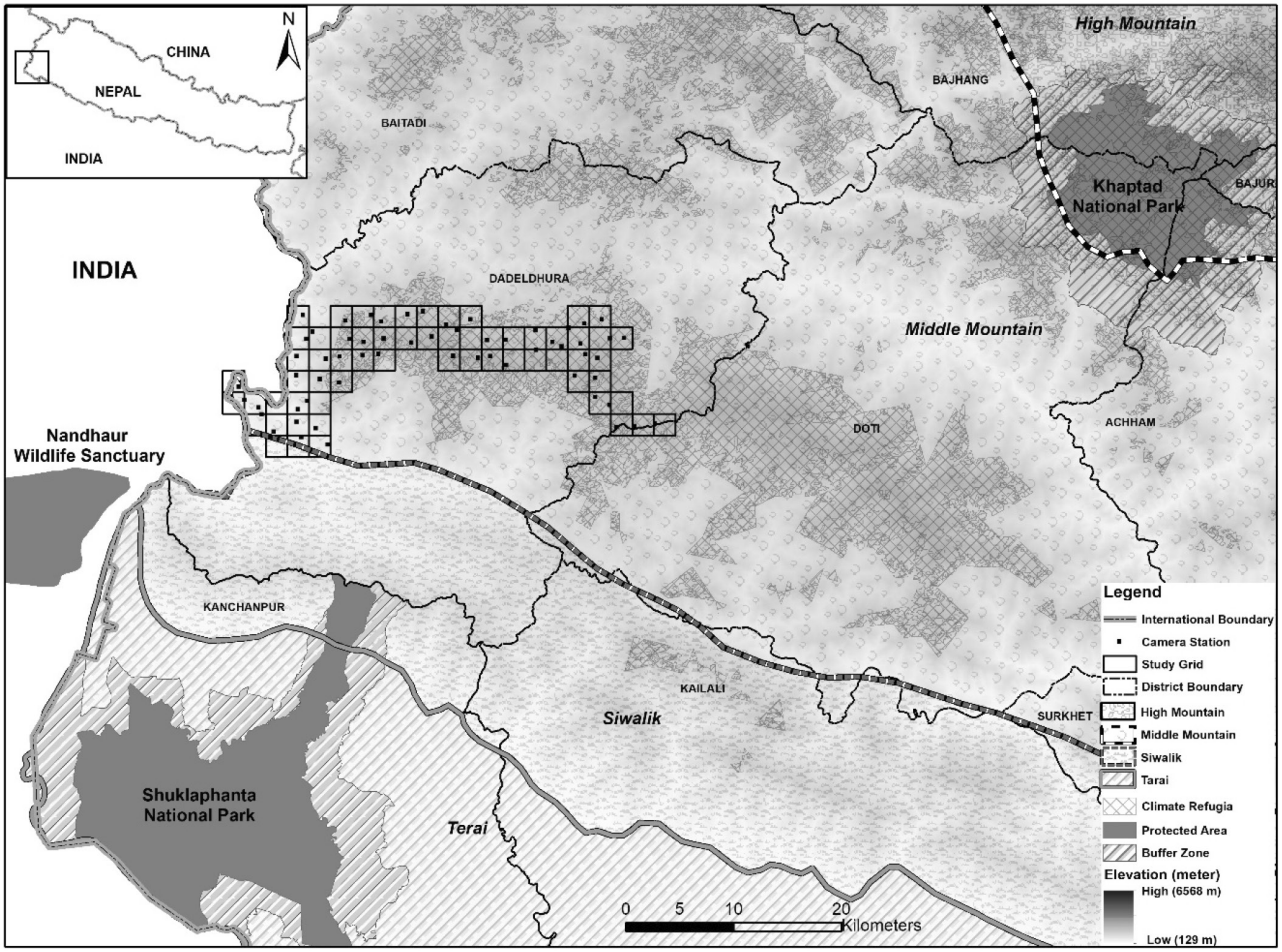
Study area showing camera trap grid (2 km by 2 km) and camera trap station (represented by black dot) including climatic microrefugia. Shuklaphanta National Park and Khaptad National Park are two nearest protected areas located in the lowland and mountain areas.

Five broad physiographic ranges are found in Nepal‐lowland (Terai, <300 m), Churia (Siwalik, 301–700 m), middle mountain (midhills, 701–2000 m), high mountains (Mahabharat, 2000–2500 m), and high Himal (<8848.86 m) (LRMP, 1986). Our monitoring efforts spanned across 250 km^2^ in the forested landscape in the north‐western mountainous areas of Nepal (Figure 1) falling within Churia and Mahabharat physiographic range. Study area lies within *Dadeldhura* district having the highest forest cover (~74.43%; 1143 km^2^) relative to other 76 districts in the country. Over 58% (~145 km^2^) of the forest area in study area has been identified as climatic microrefugia (Thapa et al., 2016). *Pinus* spps, hill sal (*Shorea robusta*) forest, and *Quercus*‐*Rhododendron* spps are major tree species found in the region. Microclimatic variables (temperature, precipitation, and humidity) vary along the elevational range (min: 418 m–max: 2547 m) suggesting variation in habitat niche. Average temperature ranges between minimum of 4°C in the month of December in winter season and maximum of 27.5°C in the month of July in monsoon season. Precipitation is estimated to be as high as 1115 mm during the monsoon to as low as 53 mm in the post monsoon season. Snowfall has been recorded at an elevation higher than 2000 m asl (above sea level) encompassing 37% of our study area.

### 2.2 Camera trap survey

**FIGURE 2:**
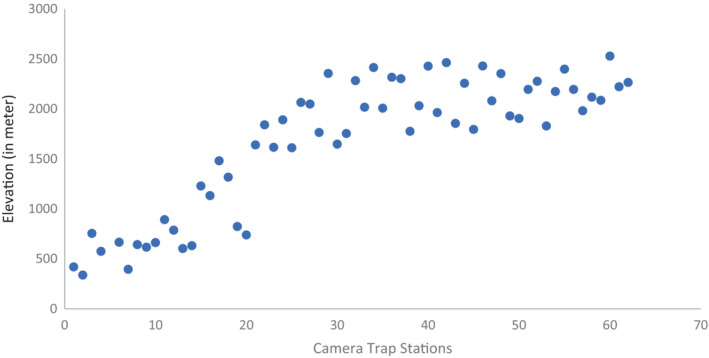
Camera trap stations (*n* = 62) deployment along the elevation range in the study area.

We conducted camera trap survey for 62 days in the post winter season from March to May 2020. We sampled 250 km^2^ of forested habitat identified as climate refugia located in Mahabharat range (Figure 1). We divided survey areas into two blocks each measuring an average 125 km^2^ as these sites were mostly inaccessible and to complete the survey in single block design due to logistical limitations. Each of the blocks were further divided into 2 × 2 km grid cells and suitable sites identified within each cell (Figure 1) for setting up camera stations. We set pairs of Cuddeback™ cameras at 62 locations and sites chosen to maximize detection and ensure ease in accessibility. Each camera trap station at each block was active for ~31 days. We followed the fourth design protocol (Karanth & Nichols, 2002) with a rotation of camera traps between the blocks sequentially covering the area of interest. We placed camera traps along the ridgelines, cliff bases, stream gorges, firelines, and trails commonly used by wild animals and people. Cameras were tied either to trees or fixed poles at the height of ~40 cm from the ground. The survey was designed to maximize capture probabilities of mammalian wildlife of varying body sizes and shoulder heights known to use the habitat traversing along similar travel routes. Cameras were placed ~3 m away on either side of the movement trail to ensure full‐body capture of mammals in the area. The inter‐trap distance between two consecutive locations was ~1.8 km (SD 0.3), with elevation ranges between 418–2457 m asl (Figure 2).

#### 2.2.1 Species identification and capture events

After careful retrieval of all images from the camera traps, we manually stored them in memory card in relevant folders. We used the Baral and Shah (2008) manual for species identification in camera trap images. We sorted the images, considering the photographs as independent events if they were 30 min or more apart, unless we could tell they were distinctly different individuals, as is commonly done in camera trap studies (Di Bitetti et al., 2006; Silver et al., 2004). We found it difficult to individually identify each wildlife captured, and thus, used capture events (number of independent photographs) per unit effort (100 trap nights) as a measure of the trapping rate or relative activity of wildlife (Kelly, 2008; Rovero & Marshall, 2009). There is a constraint associated with index‐based count (used here as the trapping rate or relative activity of wildlife) (Gopalaswamy et al., 2015; Tobler et al., 2015). Although index‐based count do not imply true abundance of the wildlife species found in the study, use of such indices has been validated in camera trap studies (Carbone et al., 2001; Rahman et al., 2021; Thapa & Kelly, 2017). We segregated identified wildlife as per the relevant taxa and IUCN's Red list of threatened species categories (IUCN, 2012).

### 2.3 Analytical method

#### 2.3.1 Species richness

We used MSOM to estimate species‐specific occurrence probabilities (Dorazio & Royle, 2005; Zipkin et al., 2010) while correcting for incomplete detection (MacKenzie et al., 2002). MSOM is known to leverage information from across the community, and rare or poorly detected species can be analyzed individually by “borrowing” data from the community (Iknayan et al., 2014). This approach seems feasible for this study as very limited information on species diversity was available from this area. We followed Bayesian approach to MSOM as used by Rahman et al. (2021) in Bangladesh for estimating species richness. Information about prior and basic code used here is available as Appendix S1. Undetected species for which no data were represented by including all‐zero encounter histories in a process known as zero augmentation (Kéry & Royle, 2009) and helped to assess the occupied range of mammal species and species richness (Broms et al., 2016; Kéry & Royle, 2020). We used data augmentation value (for undetected species) of 10 for estimating species richness and evaluated the results to explain the best possible species richness estimates for the study areas.

We estimated posterior distributions of parameters using Markov Chain Monte Carlo (MCMC) implemented in JAGS (version 3.4.0) which we called using R2Jags (Plummer, 2011) in R (R Core Development Team 3.2.2). We generated seven chains of 1,000,000 iterations after a burn‐in of 5000 iterations and thinned by 100. We assessed convergence using the Gelman‐Rubin statistic ($\hat{R}$) where values <1.1 indicated convergence (Gelman & Rubin, 1992).

Here, we estimated three parameters: (1) *ψ*_*t*_, probability of occupancy, defined as the probability of species occurring at camera trap station, (2) *p*_*s*_, probability of detection, the probability that a species is detected given that it is present, and (3) omega, overall probability of occupancy across species presented in the study areas. We presented predictive maps using mean site wise species richness estimates based on standard null model (without covariates). Due to small number of sites and low capture rate of many of the species along the location, there was issue with non‐convergence, and so, no covariates were used for assessing the factor affecting the occupancy.

#### 2.3.2 Activity pattern

We looked at daily rhythms of wildlife activity looking at the time of day, which is circular pattern, and applied circular statistics using the program ORIANA (Kovach Computing Services; Kovach, 2012). Frequency of camera trap images of a species in time reflect temporal activity of the species (Rowcliffe & Rowcliffe, 2016). We described temporal activity patterns for each camera trap recorded species by fitting a von Mises probability density distribution (Fisher, 1995) as done with tiger and its copredators in India (Karanth et al., 2017). We used the time stamp data showing the time of encounter obtained from camera trap images to compute the temporal activity pattern of species captured in the study area. All encounters were collapsed into a single 24‐h period. We segregated and assigned the temporal activity for a species to be diurnal, crepuscular (i.e., active primarily at twilight), nocturnal or cathemeral (i.e., irregularly active at any time of day or night). We also compared temporal activity of the recorded species from the study area with that from protected area in low‐lying terai (Shuklaphanta National Park) and high mountains (area along the similar range in Asia).

#### 2.3.3 Tiger distribution and connectivity in climate refuge

We carefully searched for tiger detection in camera trap locations in the present survey to ascertain its range along the northern frontier of western Himalayan range. Thapa et al. (2016) identified 140 km^2^ forest habitat as the climate microrefugia between the elevation range 418 m to 2547 m asl. With every tiger capture, we extracted ancillary information such as topographical features (elevation, aspect, and slopes). We did a comparative analysis of elevation distribution range, using a box plot, of individual camera trapped tigers with published tiger camera trap datasets from core tiger population (DNPWC & DFSC, 2018) and areas outside the protected areas (Bista et al., 2021; Subedi et al., 2021) including tiger captures from present survey.

## 3 RESULTS

We amassed 98,916 camera trap photographs in 1800 trap nights after removing 124 trap nights of camera malfunctions. Of the 24 terrestrial species recorded, 23 mammalian and one reptile species were segregated. Of the 23 mammalian species photograph captured, 10 (42%) were of high global conservation significance categorized as per IUCN's Red List of Threatened Species and 4 (15%) species are listed under the protected animal list of Nepal (Table 1). Of the mammalian species captured, 12 species of the order‐carnivora were recorded and categorized under the families including 4‐felidae, 3‐viverridae, 2‐canidae, 1‐ hyaenidae, 1‐ mustelidae, and 1‐ herpestidae. Photo images of large carnivores such as tiger, common leopard, and striped hyaena (*Hyaena hyaena*), while meso‐carnivores such as red fox (*Vulpes vulpes*) and golden jackal (*Canis aureus*) were captured. Small carnivore communities such as large Indian civet (*Viverra zibetha*), small Indian civet (*Viverricula indica*), masked palm civet (*Paguma larvata*), and leopard cat (*Prionailurus bengalensis*) were also captured. Common ungulates including barking deer (*Muntiacus muntjak*), wild boar (*Sus scrofa*), and Himalayan goral (*Naemorhedus goral*) with less common capture of sambar (*Rusa unicolor*) deer were also recorded. Indian pangolin (*Manis crassicaudata*) along with three species of primates (*Macaca mulatta*, *Semnopithecus schistaceus*, *Semnopithecus hector*) were also recorded (Table 1). Himalayan black bear (*Ursus thibetanus*) has also been recorded but outside the sampling period in the study area.

**TABLE 1:** Camera trap photo evidence with its estimated occupancy (Ψ), trapping rate, camera trapped in study area, recorded elevation range, naïve occupancy, and corresponding trapping rate for each photo captured species.

S.No	Photographic evidence	Common name	Scientific name	Camera trapped in study area	ELE (L to H) (in m)	NO	Ψ (SE(ψ))	*p* (SE(*p*))	TR	IUCN status
1	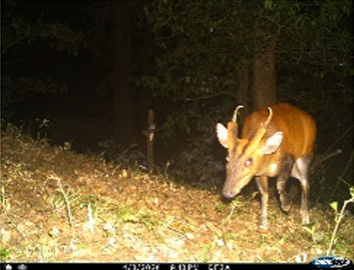	Barking Deer	*Muntiacus muntjak*	Y	418–2547	0.73	0.72 (0.07)	.07 (.01)	7.5	LC
2	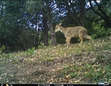	Himalayan Goral	*Naemorhedus goral*	Y	430–2547	0.31	0.39 (0.08)	.05 (.01)	2.2	NT
3	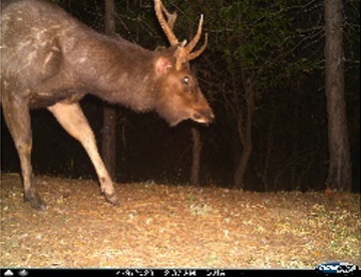	Sambar	*Rusa unicolor*	Y	1885–2134	0.06	0.08 (0.04)	.05 (.01)	0.6	VU
4	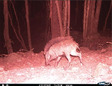	Wild Boar	*Sus scrofa*	Y	430–2547	0.60	0.77 (0.09)	.04 (.01)	4.5	LC
5	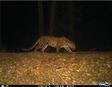	Common Leopard	*Panthera pardus fusca*	Y	418–2510	0.48	0.63 (0.08)	.05 (.01)	3.7	NT
6	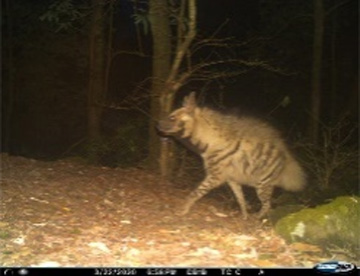	Striped Hyaena	*Hyaena hyaena*	Y	2030–2030	0.02	0.03 (0.02)	.05 (.01)	0.1	VU
7	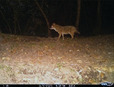	Golden Jackal	*Canis aureus*	Y	418–2547	0.50	0.49 (0.07)	.09 (.01)	6.7	LC
8	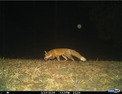	Red fox	*Vulpes vulpes*	Y	1765‐1928	0.05	0.06 (0.03)	.06 (.01)	0.6	LC
9	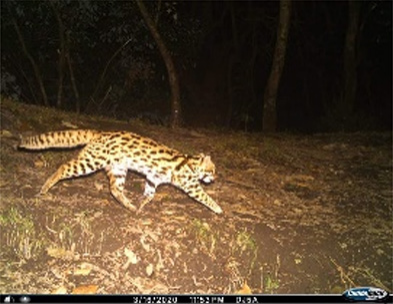	Leopard cat	*Prionailurus bengalensis*	Y	430–2547	0.47	0.56 (0.09)	.05 (.01)	3.5	LC
10	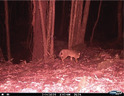	Jungle Cat	*Felis chaus*	Y	418–2326	0.18	0.21 (0.06)	.06 (.01)	1.4	LC
11	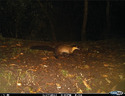	Yellow‐throated Marten	*Martes flavigula*	Y	725–2547	0.45	0.47 (0.08)	.06 (.01)	3.5	LC
12	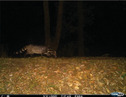	Large Indian civet	*Viverra zibetha*	Y	535–2547	0.50	0.53 (0.08)	.06 (.01)	4.8	NT
13	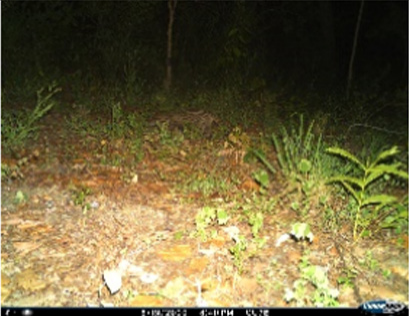	Small Indian civet	*Viverricula indica*	Y	418–418	0.02	0.03 (0.02)	.05 (.01)	0.1	NT
14	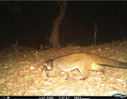	Masked palm civet	*Paguma larvata*	Y	847–2480	0.32	0.35 (0.07)	.06 (.01)	2.9	LC
15	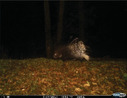	Indian crested porcupine	*Hystrix indica*	Y	2371–2371	0.02	0.02 (0.02)	.06 (.02)	0.2	LC
16	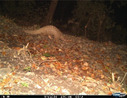	Indian pangolin	*Manis crassicaudata*	Y	530–534	0.03	0.05 (0.03)	.05 (.01)	0.1	EN
17	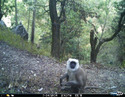	Nepal gray Langur	*Semnopithecus schistaceus*	Y	530–2480	0.06	0.08 (0.04)	.05 (.01)	0.5	LC
18	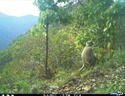	Tarai Gray langur	*Semnopithecus hector*	Y	535–2014	0.13	0.15 (0.05)	.06 (.01)	1.2	LC
19	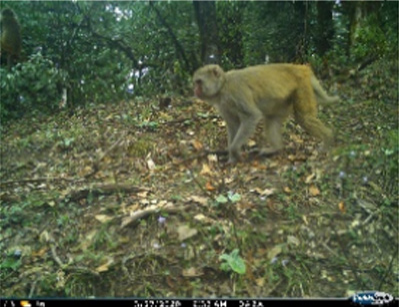	Rhesus Macaque	*Macaca mulatta*	Y	2371–2371	0.02	0.02 (0.02)	.06 (.02)	0.2	LC
20	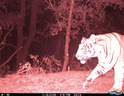	Tiger	*Panthera tigris*	Y	2511–2511	0.02	0.03 (0.02)	.06 (.02)	0.1	EN
21	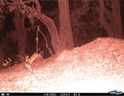	Indian hare	*Lepus nigricollis*	Y	418–2547	0.27	0.28 (0.06)	.08 (.01)	3.5	LC
22	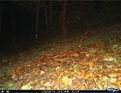	Mouse	*Order: Rodentia*	Y	430–2371	0.13	0.13 (0.05)	.06 (.01)	0.8	LC
23	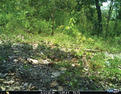	Small Asian Mongoose	*Herpestes javanicus*	Y	418–418	0.02	0.03 (0.02)	.05 (.01)	0.1	LC
24	NA	Spotted deer	*Axis axis*	N	–	–	–	–	–	LC
25	NA	Himalayan black bear	*Ursus thibetanus*	N	–	–	–	–	–	VU
26	NA	Himalayan serow	*Capricornis thar*	N	–	–	–	–	–	VU
27	NA	Dhole	*Cuon alpinus*	N	–	–	–	–	–	EN
28	NA	Assamese monkey	*Macaca assamensis*	N	–	–	–	–	–	NT
29	NA	Red panda	*Ailurus fulgens*	N	–	–	–	–	–	EN
30	NA	Clouded Leopard	*Neofelis nebulosa*	N	–	–	–	–	–	VU

*Note*: IUCN species conservation status: EN, endangered; VU, vulnerable; NT, near threatened; LC, least concern; ND, no. of detection; NO, Naïve Occupancy; SE, standard error; TR, trapping rate; Elev, elevation; L, low; H, high; Y, Yes; N, No; NA, not available; S.No, Serial Number.

### 3.1 Occupancy and species richness

**FIGURE 3:**
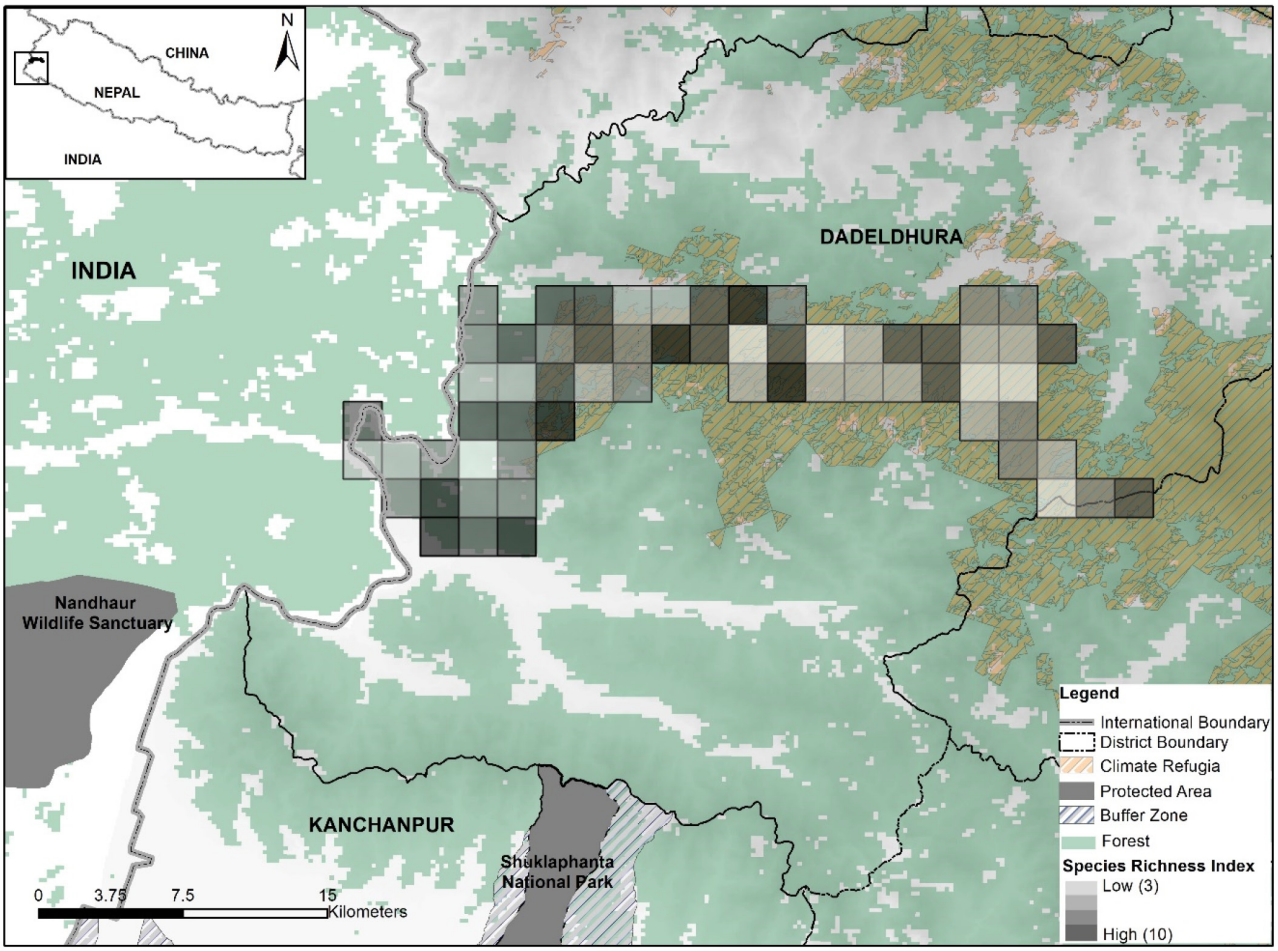
Site level species richness estimates variablity along the identified climatic microrefugia in the western part of Nepal.

Species‐specific estimate of occupancy, ψ(SE(ψ)) via MSOM, ranged from 0.77 (0.09) for wild boar to least 0.02 (0.02) for Indian crested porcupine (*Hystrix indica*) with mean occupancy across the mammalian community was 0.87 (0.09) (Table 1). Our estimated detection probabilities $\hat{p}$(SE ($\hat{p}$)) was highly variable among species ranging from .09 (.01) for golden jackal to least .04 (.01) for wild boar. MSOM estimated the species richness with unknown species richness to have a median value of 30 species (95% CI: 25–34). Among all sites, our camera‐station specific estimates of mammalian richness ranged from 3 (95% CI: 1–5) to 10 (95% CI: 9–12) with a mean estimated richness per camera trap station of 6 (Figure 3).

### 3.2 Temporal activity of the wildlife and human across the refugia

**FIGURE 4:**
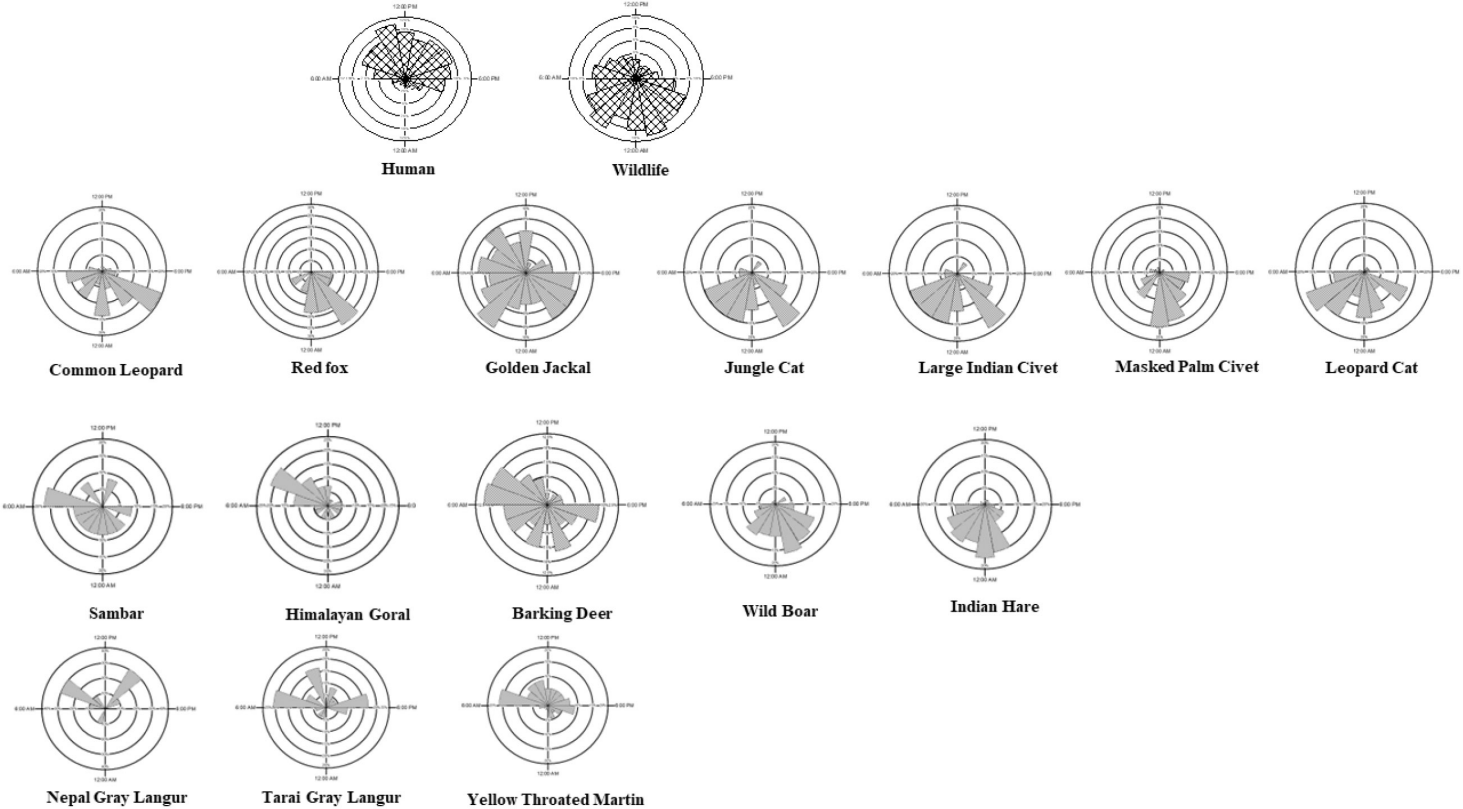
Proportion of encounters of the human and wildlife during daytime (06:00–17:59 h; hashed wedges above half circle) and nighttime (18:00–05:59 h; hashed wedges below half circle) in climatic microrefugia. Rose diagrams were generated using ORIANA (Kovach, 2012). Each plot is divided into 24 h, with percentage of detections in each hour on the response axis.

High human presence has been recorded along the forested area with probability of occupancy estimated at 0.92 (SD 0.03) with their activity identified during day time (Figure 4). Temporal interactions between wildlife and human were inversely correlated with wildlife depicting an overall crepuscular and nocturnal activity (active at dawn and dusk, Figure 4). All the carnivores species recorded in the study areas were found to be active during the night except golden jackal, which was active most of the time showing cathemeral behavior (Figure 4). Among the herbivores, highly abundant barking deer was active day and night including sambar‐largest of the deer species recorded in the study area. The Tarai gray langur and Nepal gray langur‐showed diurnal temporal pattern within the study areas. We found variations in temporal activity patterns among the recorded species in the study area in comparison with those in other protected areas. More than half (~50%–71%) of the recorded species were either strictly nocturnal or cathemeral (Table 2).

**TABLE 2:** Comparison of temporal activity patterns of recorded species with the nearest protected areas in lowland areas (Shuklaphanta National Park or other lowland protected areas) and available published data from mountain protected areas.

Species	Temporal activity in study area	Temporal activity in lowland area in ShNP	Temporal activity from mountain protected areas	References
Common leopard	Nocturnal	Nocturnal	Cathemeral in Manas National Park, India	Bhatt et al. (2021)
Red fox	Nocturnal	Not Recorded	Unknown	–
Golden jackal	Cathemeral	Diurnal	Cathemeral in MCA	Katuwal and Dahal (2013)
Jungle cat	Nocturnal	Crepuscular	Nocturnal in churia habitat in Chitwan district	DNPWC unpublished data
Large Indian civet	Nocturnal	Nocturnal	Nocturnal in ACA	Appel et al. (2013)
Masked palm civet	Nocturnal	Not Recorded	Nocturnal in Houhe National Nature Reserve, China	Zhou et al. (2014)
Sambar	Cathemeral	Nocturnal	Nocturnal in Shivpuri National Park, Nepal	DNPWC unpublished data
Himalayan goral	Diurnal	Not Recorded	Nocturnal in Sikkim, India	Bhattacharya et al. (2012)
Barking deer	Cathemeral	Diurnal in CNP	Cathemeral in Manas National Park, India	Bhatt et al. (2021)
Wild boar	Nocturnal	Diurnal in CNP	Diurnal, in Manas National Park, India	Bhatt et al. (2021)
Indian hare	Nocturnal	Crepuscular to Nocturnal	Diurnal in Deccan Peninsula, India	Behera et al. (2022)
Nepal gray langur	Diurnal	Not Recorded	Diurnal in Langtang National Park	Sayers and Norconk (2008)
Tarai gray langur	Diurnal	Diurnal	Diurnal outside protected in eastern hills in Dharan	Tamang et al. (2020)
Yellow‐throated marten	Diurnal	Diurnal	Crepuscular in Makalu Barun National Park	Basnet and Rai (2020)

Abbreviations: ACA, Annapurna Conservation Area; CNP, Chitwan National Park; DNPWC, Department of National Parks and Wildlife Conservation; MCA, Manaslu Conservation Area; ShNP, Shuklaphanta National Park.

### 3.3 Detection of tiger in climatic microrefugia

**FIGURE 5:**
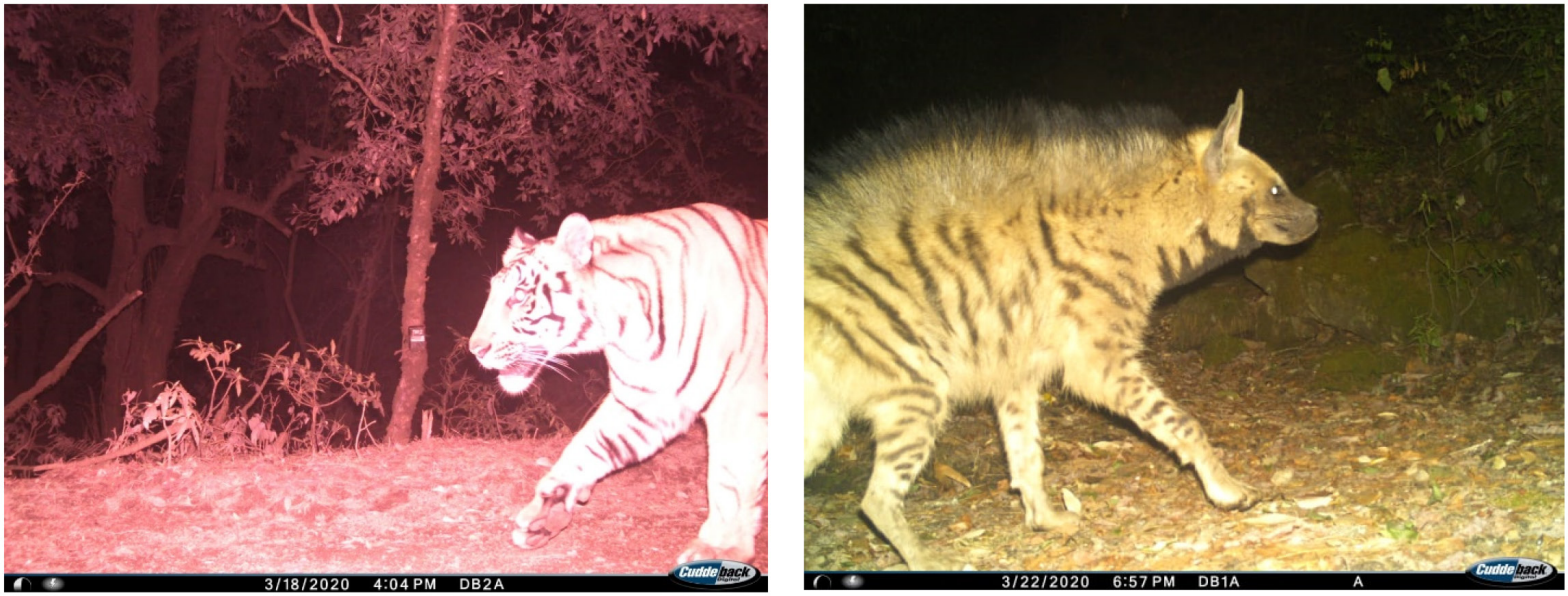
Photographic evidence of Tiger (left) and striped hyaena (right) captured at an elevation of 2511 m and 2070 m in forested habitat of Dadeldhura District in Western Himalaya in Nepal. Site has been identified as climatic microrefugia (Thapa et al., 2016).

**FIGURE 6:**
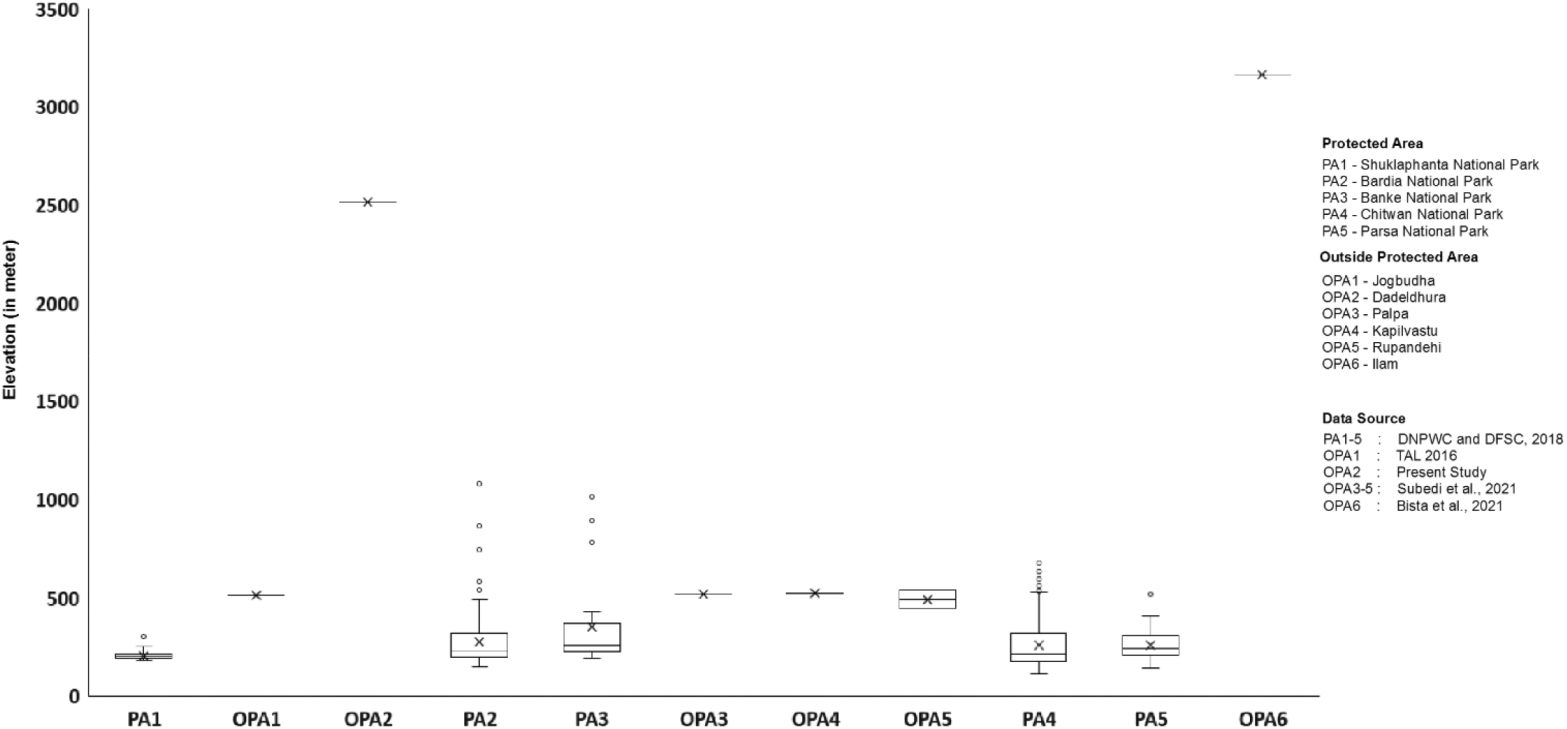
Box plot (with median values and cross marker represent mean value along with outliers represented in dots) showing the distribution of altitudinal gradient of camera trapped tiger population along the various sites (west to east) in Nepal. Elevation data on Shuklaphanta National Park (PA1), Bardia NP (PA2), Banke NP (PA3), Chitwan NP (PA4), and Parsa NP (PA5), Jogbudha (OPA1) (TAL, 2016) and Dadeldhura (OPA2) (present survey), Palpa (OPA3), Kapilvastu (OPA4), and Rupendehi (OPA5), and Ilam (OPA6).

From the stacks of images representing 24 terrestrial species, we recorded the photographic evidence of tiger (probably male) with two unique detections at 16:00 p.m. at elevation of 2511 m asl on the western aspect of Mahabharat range in western Nepal (Figure 5). Within the study area, tiger occupancy is estimated at 0.03 (0.02). Comparatively, 72% of tiger captures were recorded in terai along the elevation range <300 m, 26% in Churia between 300–700 m and rest 2% above 700 m (Figure 6). Among the recorded tiger locations in Nepal, approximately 14% falls within habitat identified as climate microrefugia.

## 4 DISCUSSION

In Nepal, the Dadeldhura hill forest ranges have been identified as a climatic microrefugia and as biologically diverse and ecologically significant areas with high species richness and occupancy. Our camera trap assessment of climate‐refuges in Nepal (Thapa et al., 2016) is the first of its kind to focus on mammalian species diversity and distribution. Major findings of the study have been (a) mammalian species richness estimated at 30 species and mean occupancy probability across the mammal community at 0.87 with variability along the captured locations/sites; (b) many of the wildlife species exhibited nocturnal rather than diurnal temporal activity; and (c) detection of tiger and hyaena at highest altitudinal range in the western Himalayan range in Nepal with possible connectivity to Shuklaphanta National Park in the south (Figure 1).

### 4.1 Species richness in climate microrefugia

Our study is the first to focus on mammalian diversity outside the protected areas system in Nepal and in habitat identified as climatic microrefugia (Thapa et al., 2016). Our estimates are based on MSOM with species richness at 30 closes to true mean with narrow confidence interval. This study provides a baseline on faunal diversity in an area outside protected areas having the highest forest cover including for sites identified as climate microrefugia. Result represents species richness outside protected areas system which is comparable to studies adopting similar analytical technique via MSOM in Bhutan (Penjor et al., 2021) and Bangladesh (Rahman et al., 2021). Dadeldhura forest habitat exhibited an ecotone harboring mammals that represent subtropical terai forest to temperate and subalpine forest‐dwelling species from Tarai gray langur, small Indian civet, jungle cat, striped hyaena, Indian pangolin to red fox, Himalayan black bear, Nepal gray langur, and Himalayan goral at an altitudinal gradient between 418 m and 2547 m. Striped hyaena seemed to roam significantly more frequently in the hilly areas (around 200–400 m asl) (Bhandari et al., 2020) and their presence has been reported upto 1750 m (Majupuria & Majupuria, 2006). The present study photo captured hyaena's presence at an elevation of 2030 m (Figure 5)—the first record of this species at this altitude.

Species such as spotted deer, Himalayan black bear, dhole, Himalayan serow, Assamese macaque have a possible range within study areas (Baral & Shah, 2008) and could be pseudo absent during the effective sampling period for this study. Dadeldhura hill forests provide suitable habitats with a potential distribution of arboreal species such as red panda (Thapa et al., 2020). Thus, this species could have been missed due to layout of the camera trap that focused on ground‐dwelling faunas. Comparable mean camera‐specific species richness (~6.19) in Dadeldhura forest hills and Churia habitat (~5.72) in Chitwan National Park (Rich et al., 2014) was found, along with estimated species richness of 30 species in Dadeldhura hills versus the observed richness of 37 species in Shuklaphanta National Park (Poudyal et al., 2019). Clouded leopard (*Neofelis nebulosa*) has been reported in Api‐Nampa Conservation Area and Khaptad National Park in the western region; however, species westward distribution needs to be verified (Ghimirey & Acharya, 2017).

Forest cover (~1143 km^2^) within Dadeldura district is highest in the western region in comparison with other districts. This, along with diverse vegetation types ranging from subtropical to subalpine forests providing suitable habitats for range of species, could be a reason for high forest‐dwelling species diversity. Second, recovery of primary forest tract over a period after deforestation (estimated at ~1.1% per year) could be a reason for recolonization of species, possibly rewilding (Soulé & Noss, 1998), ensuring comparable species richness to nearby protected areas. Since late 70s, most of forest stands along the periphery of settlements in the districts have been handed over to the community as community forest where by forest resources are used and managed by the communities. The community forestry (CF) program is well institutionalized in Nepal with handing over the management of the forest to the communities living nearby (Acharya, 2002). A well‐coordinated action in CFs periodic forest operational plan can also prioritize the species protection action. Forest hill tract provides contiguous habitat between protected areas in Nepal and India, and thus may offer opportunity to manage the habitat as a corridor for facilitating ecological processes such as species mediate dispersal, additional climate safe space, and its persistence in extreme climate condition in the low‐lying areas. Division Forest Directorate of the Provincial Government has pushed for the proposal to manage the present study site as an area of high conservation value and could be declared as forest conservation area. Provincial Forest Ministry has accordingly been providing annual budgetary support to Division Forest Office Dadeldhura to manage the proposed forest conservation area. Currently, proposed forest conservation area has mixed management regime as government managed forest and community forest. Both management regime follows an approach where by forest resource management and utilization for human consumption follows forest operation plan. Government of Nepal shall declare such forest protection area as biological corridor to protect wildlife as per Government of Nepal, Forest Act 2019‐Clause 16. With evidence of extreme climate conditions such as drought, flooding, and rising temperatures, contiguous habitats such as these can provide refuge for wildlife (species occupancy Psi = 0.87 in the present study) dispersing from lowland areas such as Shuklaphanta National Park, Nepal to Nandhaur Wildlife Sanctuary, Champawat, and Boom Forest range across the border in India. The applicability of MSOM utilizing the camera trap data provided very useful and important metrics in a single framework for faunal assessment.

### 4.2 Temporal activity of wildlife and human

Majority of the wildlife species, and especially carnivores, were found to be active during the nighttime. Leopards and leopard cats were found to be active during the nighttime, as was found in Langtang National Park (Can et al., 2020) with similar landscape heterogeneity. Barking deer (both diurnal and nocturnal) and wild boar (mostly nocturnal) were both detected through the elevational gradient and represents the principal prey for mid‐sized carnivores such as common leopard and hyaena. From our data and other studies in lowland Nepal (Poudyal et al., 2019) and Thailand (Lynam et al., 2013), leopard cats are strongly nocturnal. Himalayan goral—a goat species—largely confined in Churia (Thapa & Kelly, 2017) and high mountains upto 3200 m asl (Katuwal et al., 2013) were largely active during day time, while being less active at night and with activity peak higher in the morning time in our study area in concordance with work from Bhattacharya et al. (2012) and could be potential prey for the common leopard. Yet, Himalayan goral's preference for steeper slopes habitat (Bhattacharya et al., 2012) and thereby energy requirement for predation requires further investigation for their contribution in the carnivore diets. Of the four species of primates found in Nepal (Chalise, 2004), three species of primates were wide spread and active during day time in concordance with similar studies elsewhere (Sun et al., 2020). We found high reported case of negative interaction between humans and wildlife, especially wild boar (personal communication: Mr Dipendra KC, Assistant Division Forest Officer‐ Dadeldhura Division Forest Office); their temporal activity suggested crop raiding during the nighttime, in concordance with our results. Yellow‐throated marten is mostly solitary, and our results corroborate the primarily diurnal activity pattern reported by earlier studies (Appel et al., 2013; Grassman et al., 2005). Gaynor et al. (2018) argued on global increase of nocturnality among wildlife in human‐dominated areas demonstrating the high degree of behavioral plasticity of animals in a human‐altered world. Our study also supports this global notion as almost 50%–71% of the species were found to be either strictly nocturnal or cathemeral in the study area.

### 4.3 Detection of tiger in high altitude habitat

In the Indian‐subcontinent, tigers (*Panthera tigris tigris*) are known to be generalists and occur in a multitude of habitat types featuring a range from saline mangrove forests in India (Roy et al., 2016) to floodplains in Nepal (Thapa et al., 2017) and going up to 4400 m asl in alpine forest habitat in Bhutan (Tempa et al., 2019). Tiger recorded at an elevation of 3100 m asl in the eastern Himalayan range remains the highest altitudinal record in Nepal (Bista et al., 2021). Detection of tiger at 2511 m asl indicates forested habitat as an exploratory range‐for dispersing tigers at high altitude in the western Himalayan range in Nepal. Forested habitats provide strategic connectivity between the source/sink populations between Nepal and India in the eastern region. Tiger has been recorded at a higher elevation at 3274 m asl near Askot Wildlife Sanctuary in 2016 (Bhattacharya & Habib, 2016) and Kedarnath Musk Deer Sanctuary in 2019 (Pawar et al., 2020) in India which are the nearest recorded range along the highland transboundary areas in the western Nepal. As of now, Shuklaphanta National Park (24 km aerial distance) in Nepal and/or Nandhaur Wildlife Sanctuary in India (32 km aerial distance) remain proximate core populations to the carnivore's probable origin. More camera trap survey is needed to ascertain where the tiger originates in relation to the source and sink population available and/or relic population given good cover and fair prey population in the Dadeldhura hill forest.

The considerable extent of these forest habitats can sustain tigers given the presence of prey species with varying trapping rate and occupancy (ψ) such as barking deer (7.5, 0.72), Himalayan goral (2.2, 0.39), wild boar (4.5, 0.77), sambar (0.6, 0.08), and primates (0.2–1.2; 0.02–0.15). Prey such as wild boar has been identified as key species of breeding tiger population at high altitude in Bhutan (Tempa et al., 2019). Yet, the poaching threat has been eminent with potential evidence such as photographs of people carrying guns for hunting. A strong conservation program prioritizing the protection is anticipated for the survival of wildlife. Wildlife Crime Control Bureau (WCCB) brings in collective actions among the law enforcement agencies in Nepal to improve protection measures strengthening poaching control and disrupting illegal wildlife trade nexus in the respective district (Aryal et al., 2017). Dadeldhura district has been identified 30th WCCB district cell in Nepal.

Tiger distributional range along the higher elevation in the eastern and the western Himalayas have been continuing with the capture of the tiger along various locations over time (Bhattacharya & Habib, 2016; Bista et al., 2021; Pawar et al., 2020; Umariya et al., 2021). Tiger‐bearing protected areas lies along the altitudinal variation (<700 m) through terai, churia, and middle mountain physiographic zones in Nepal. Nepal tiger‐bearing protected area altitude ranges between 112 m asl in Chitwan/Parsa National Parks to high as 1576 m asl in Bardia National Park. Thus, tiger distribution above cutoff 2000 m asl represents habitat in the physiographic zone—high mountains and above—can be typified as high‐altitude tiger range in Nepal. This highlights the importance of Churia, middle, and high mountains for tiger and their prey base conservation (including other wildlife) in the face of changing climate with due importance to larger climatic macrorefugia (~47,400 km^2^) sites identified in these physiographic zones. More camera trap survey with protocol as defined here are anticipated in high‐altitude habitats—focusing on high mountains—also known as the Mahabharat range. Nepal has been successful in achieving doubling of wild tigers in its core areas; additional efforts to identify habitat suitability outside protected areas regime including in Mahabharat range forest areas by intervening in forest and wildlife management seems pertinent in days to come.

## AUTHOR CONTRIBUTIONS

**Kanchan Thapa:** Conceptualization (lead); data curation (equal); formal analysis (lead); funding acquisition (supporting); investigation (lead); methodology (lead); project administration (supporting); resources (supporting); software (lead); supervision (lead); validation (lead); visualization (lead); writing – original draft (lead); writing – review and editing (equal). **Samundra Ambuhang Subba:** Conceptualization (equal); data curation (equal); formal analysis (equal); funding acquisition (equal); investigation (equal); methodology (equal); project administration (equal); resources (equal); software (equal); supervision (equal); validation (equal); visualization (equal); writing – review and editing (equal). **Gokarna Jung Thapa:** Formal analysis (supporting); investigation (supporting); methodology (supporting); software (lead); visualization (lead); writing – review and editing (supporting). **Karun Dewan:** Investigation (supporting); project administration (equal); writing – review and editing (supporting). **Bishnu Prasad Acharya:** Conceptualization (equal); investigation (supporting); project administration (lead); resources (supporting); supervision (supporting); writing – review and editing (supporting). **Dabal Bohara:** Project administration (supporting); resources (supporting); supervision (supporting); writing – review and editing (supporting). **Suman Subedi:** Conceptualization (supporting); funding acquisition (supporting); project administration (lead); supervision (lead); writing – review and editing (supporting). **Madhuri Thapa Karki:** Project administration (supporting); resources (supporting); supervision (supporting); writing – review and editing (supporting). **Bharat Gotame:** Funding acquisition (equal); project administration (supporting); resources (supporting); supervision (supporting); writing – review and editing (supporting). **Gautam Paudel:** Project administration (supporting); resources (supporting); supervision (supporting); writing – review and editing (supporting). **Shiv Raj Bhatta:** Conceptualization (supporting); funding acquisition (supporting); project administration (supporting); resources (supporting); supervision (supporting); writing – review and editing (supporting). **Shant Raj Jnawali:** Supervision (supporting); writing – review and editing (supporting). **Sabita Malla:** Data curation (supporting); funding acquisition (supporting); investigation (supporting); supervision (supporting).

## CONFLICT OF INTEREST

None.

## Supporting information


Appendix S1

## Data Availability

Data related to this article are deposited in Dryad and are available via the following link https://doi.org/10.5061/dryad.9kd51c5m1.
