# Supplementary material for: Wildlife in climate refugia: Mammalian diversity, occupancy, and tiger distribution in the Western Himalayas, Nepal
Source: Ecol Evol. 2022 Dec 8;12(12):e9600. doi: 10.1002/ece3.9600 (PMC9731921; doi:10.1002/ece3.9600)
Supplement: Supplementary file 1 — Appendix S1 [file ECE3-12-e9600-s001.docx]

**Information on Prior and Code used in MSOM extracted from Rahman et al., 2021.**

Paper used the Multi Species Occupancy Model for estimating species richness and multispecies occupancy in the study areas. We followed the Basic model used in the analysis provided by Rahman *et. al.* (2020) which followed Kery and Royle (2016) for species richness estimation following analysis techniques from Broms et al. (2016).

Following and extracting the informative priors from Broms et al. (2016), here in the community level analysis, the total species richness is unknown, and we have used the analysis to derive required biodiversity metrics such species richness and combined species occupancy for the study area. Since the species richness is unknown, one would augment the model with a Bernoulli variable, *w*_i_, that indicates whether or not a species exists in the assemblage of interest

w_i_ ∼Bernoulli(π).

If species i was ever detected during the study, then w_i_ = 1; otherwise it is inferred from the model similar to how the unknown, true occurrences, z_ij_, are estimated. Species richness is then a derived quantity, $N=\sum_{i=1}^{M} w_{i}$ , where M is an upper limit forspecies richness that is specified a priori. Based on the literature review, M is used here 10. MSOM code for the analysis was provided by Hasan et al (2020) upon a request. Basic model includes-

model{

#Define prior distributions for community-level model parameters

omega ~ dunif(0,1)

u.mean ~ dunif(0,1)

mu.u <- log(u.mean) - log(1-u.mean)

v.mean ~ dunif(0,1)

mu.v <- log(v.mean) - log(1-v.mean)

tau.u ~ dgamma(0.1,0.1)

tau.v ~ dgamma(0.1,0.1)

for (i in 1:(n+nzeroes)) {

#Create priors for species i from the community level prior distributions

w[i] ~ dbern(omega)

u[i] ~ dnorm(mu.u, tau.u)

v[i] ~ dnorm(mu.v, tau.v)

#Create a loop to estimate the Z matrix (true occurrence for species i

#at point j.

for (j in 1:J) {

logit(psi[j,i]) <- u[i]

mu.psi[j,i] <- psi[j,i]*w[i]

Z[j,i] ~ dbern(mu.psi[j,i])

#Create a loop to estimate detection for species i at point k during #sampling period k.

for (k in 1:K[j]) {

logit(p[j,k,i]) <- v[i]

mu.p[j,k,i] <- p[j,k,i]*Z[j,i]

X[j,k,i] ~ dbern(mu.p[j,k,i])

} } }

#Sum all species observed (n) and unobserved species (n0) to find the

#total estimated richness

n0 <- sum(w[(n+1):(n+nzeroes)])

N <- n + n0

#Finish writing the text file into a document called basicmodel.txt

}

Reference:

Broms, K. M., Hooten, M. B., & Fitzpatrick, R. M. (2016). Model selection and assessment for multi‐species occupancy models. *Ecology, 97*(7), 1759-1770.

Marc Kéry, J. Andrew Royle (2016). Applied Hierarchical Modeling in Ecology Analysis of Distribution, Abundance and Species Richness in R and BUGS, vol. 1.

Rahman, H. A., McCarthy, K. P., McCarthy, J. L., & Faisal, M. M. (2021). Application of Multi-Species Occupancy Modeling to assess mammal diversity in northeast Bangladesh. *Global Ecology and Conservation, 25*, e01385.
